# 10% Urea Ointment Plus Skin Hygiene Advice Versus Skin Hygiene Advice Alone for Prophylaxis of Radiation-Induced Skin Reactions in Cancer Patients: A Quasi-experimental Study

**DOI:** 10.7759/cureus.117709

**Published:** 2026-10-09

**Authors:** Nazirum Mubin, Dipok Saha, Tannima Adhikary, Rukhshana Rabbani, Aliya Shahnaz

**Affiliations:** 1 Department of Radiotherapy, Dhaka Medical College Hospital, Dhaka, BGD; 2 Department of Radiotherapy, Sirajganj Medical College Hospital, Sirajganj, BGD; 3 Clinical Oncology, Imperial College London, London, GBR

**Keywords:** non-randomized study, quasi-experimental study, radiation dermatitis, radiation-induced skin reactions, radiotherapy, supportive care, urea ointment

## Abstract

Background

Radiation-induced skin reactions (RISRs) are a frequent acute toxicity of radiotherapy that can cause pain, impair quality of life, and interrupt treatment. No topical agent is established for their prevention in Bangladesh, and the value of inexpensive 10% urea ointment is unknown.

Methodology

This prospective, quasi-experimental, open-label study was conducted in the Department of Radiotherapy, Dhaka Medical College Hospital, Bangladesh, from December 1, 2021 to May 31, 2022. A total of 64 patients receiving external-beam radiotherapy were assigned in equal numbers by a predetermined odd-even sequence to twice-daily 10% urea ointment plus skin hygiene advice from the first fraction until 14 days after completion, or to the departmental 2022 practice of hygiene advice alone. RISRs and skin pain were assessed by Radiation Therapy Oncology Group (RTOG) criteria and a visual analog scale (VAS) weekly during treatment and at two and four weeks afterward. The primary outcome was RISR incidence; secondary outcomes were its onset, duration and severity, skin pain, analgesia use, and all-cause unplanned radiotherapy interruption. Exact tests were used, with exploratory adjustment by modified Poisson regression.

Results

Each arm comprised 32 patients. RISRs occurred in 15/32 (46.9%) of the urea arm and 24/32 (75.0%) of the hygiene advice arm (risk ratio (RR) = 0.62, 95% confidence interval (CI) = 0.41-0.95) and were first documented later with urea (log-rank p = 0.024). Severe (grade ≥3) reactions (18.8% versus 31.2%) and median duration (15.0 versus 21.5 days, p = 0.082) did not differ significantly. Analgesia was required by 9/32 (28.1%) and 19/32 (59.4%) (RR = 0.47, 95% CI = 0.25-0.88), and all-cause unplanned interruption, with unrecorded reasons, occurred in 4/32 (12.5%) and 22/32 (68.8%) (RR = 0.18, 95% CI = 0.07-0.47); no patient discontinued radiotherapy, and weekly VAS scores never differed (all p ≥ 0.068). After adjustment, the associations with incidence (RR = 0.67, 95% CI = 0.45-0.98) and interruption (RR = 0.29, 95% CI = 0.11-0.80) persisted, but that with analgesia did not (RR = 0.57, 95% CI = 0.28-1.17).

Conclusions

In this small non-randomized study, adding twice daily 10% urea ointment to skin hygiene advice was associated with fewer and later documented RISRs. The lower rate of all-cause interruption needs cautious interpretation because its reasons were not recorded, and lower analgesia use did not persist after adjustment. Because allocation was deterministic and the groups differed at baseline, these non-randomized estimates are not causal and do not establish superiority over other moisturizers. Urea is a low-cost candidate warranting confirmation in an adequately powered randomized multicenter trial.

## Introduction

Radiotherapy is a cornerstone of cancer management and is required by roughly half of all patients during their disease course [1]. Radiation-induced skin reactions (RISRs) remain among its most frequent acute toxicities and develop in the great majority of irradiated patients [2,3]. They usually appear within one to four weeks of starting treatment, may take a further two to four weeks to resolve, and range from faint erythema and dry desquamation to painful moist desquamation, ulceration, and occasional secondary infection [3,4].

Each fraction depletes proliferating basal keratinocytes, damages the dermal microvasculature, and recruits inflammatory cells, so that cumulative injury gradually outstrips the capacity of the skin for renewal [4]. Severity depends on the dose per fraction, total dose, irradiated volume, treatment site, use of bolus, concurrent chemotherapy, and individual radiosensitivity [3,5]. Severe RISRs impair quality of life and can force interruption of radiotherapy, prolonging overall treatment time and potentially compromising local tumor control [2,6], so effective prophylaxis is an important goal of supportive care.

Many agents have been evaluated for the prevention of RISRs, including topical corticosteroids, hyaluronic acid preparations, barrier films and dressings, photobiomodulation, proprietary emulsions such as StrataXRT, and even bacterial decolonization of the skin [7-13]. The 2023 Multinational Association of Supportive Care in Cancer consensus recommends selected specific interventions for prevention, including the topical corticosteroids mometasone and betamethasone, Hydrofilm, and, in patients with breast cancer, Mepitel film and photobiomodulation, each with population-specific qualifications [7]. The certainty of evidence for most agents nevertheless remains low, recommendations differ between guidelines, and no single regimen is universally accepted [14-18]. Much of this evidence comes from breast cancer populations [8], and some recommended products are costly or difficult to obtain where resources are limited. Among simple measures, keeping the irradiated skin adequately hydrated has a strong physiological rationale, and moisturizers and other topical non-steroidal agents have been shown to reduce acute reactions and improve skin-related quality of life [19-21].

This burden is especially relevant in Bangladesh, where cancer incidence is rising, radiotherapy capacity is limited, and most patients attending public cancer centers are of low socioeconomic status [22,23]. Out-of-pocket spending on cancer care is a recognized source of financial hardship, so the cost of managing skin reactions is a real barrier to completing curative radiotherapy [24,25]. Urea is a natural humectant of the stratum corneum that binds water and supports the epidermal barrier, making it a plausible and inexpensive candidate. The closest previous study, by Pardo Masferrer et al., evaluated a lotion containing 3% urea combined with polidocanol and hyaluronic acid in 98 women with breast cancer in a prospective observational design and compared them with 174 historical controls; intensive use was associated with a higher proportion of patients remaining free of radiodermatitis (27.6% versus 15.5%) [26]. That study did not test a 10% urea preparation, did not use concurrent controls, did not include head and neck or pelvic tumors treated on a cobalt unit, and its comparator differed from the skin hygiene advice without any topical agent that constituted routine practice in our department in 2022 [5,16]. To our knowledge, no prospective study of RISR prophylaxis has been reported from Bangladesh. The primary objective of this study was therefore to compare the incidence of RISRs between patients who added twice daily 10% urea ointment to this hygiene advice and patients who received the hygiene advice alone. Secondary objectives were to compare the onset, duration, and severity of reactions graded by the Radiation Therapy Oncology Group (RTOG) criteria, skin pain, analgesia use, and all-cause unplanned interruption of radiotherapy.

## Materials and methods

Study design and setting

This prospective, quasi-experimental study was conducted in the Department of Radiotherapy, Dhaka Medical College Hospital, Dhaka, Bangladesh, over a six-month period between December 1, 2021, and May 31, 2022. The study period included participant recruitment, delivery of radiotherapy, and completion of all post-radiotherapy follow-up assessments. Recruitment was therefore closed sufficiently early to ensure that the final enrolled participant could complete radiotherapy and the four-week post-radiotherapy assessment within the study period.

Participants

Eligible patients were adults with histologically confirmed cancer scheduled for external-beam radiotherapy on a cobalt-60 machine for at least four weeks, with the treatment field including the skin. Patients were excluded if they had skin reactions from other causes (prior chemotherapy or radiotherapy, or pre-existing bacterial or fungal infection), atrophic skin changes, hypersensitivity to urea, or diabetes mellitus, were receiving concurrent chemoradiation, or were unable to attend follow-up. Socioeconomic status was recorded at enrollment on the study proforma from the self-reported monthly household income of the participant’s family. Households earning below BDT 15,000 per month were classified as poor, those earning BDT 15,000 to BDT 50,000 as middle class, and those earning above BDT 50,000 as upper class, and the same bands were applied to every participant throughout the study.

Sample size

The sample size was based on planning assumptions derived from departmental clinical experience before the study, namely, an incidence of RISR of 56% with hygiene advice alone and 22% with a urea-containing prophylactic preparation. Because no formal local audit data are available for publication, these figures are reported as planning assumptions rather than measured rates. The study of Pardo Masferrer et al. supported the plausibility of a reduction with urea prophylaxis but not this particular effect size, because the radiodermatitis-free proportions of 27.6% and 15.5% reported in that study correspond to radiodermatitis incidences of 72.4% and 84.5% [26]. The number required per arm was obtained from the normal approximation for the comparison of two independent proportions, using the formula below.

\[ n = \frac{\left[ Z_{1-\alpha/2}\sqrt{2\bar{p}(1-\bar{p})} + Z_{1-\beta}\sqrt{p_1(1-p_1)+p_2(1-p_2)} \right]^2}{(p_1-p_2)^2} \]

Here, n is the number of participants required in each group, p1 = 0.56 and p2 = 0.22 are the anticipated proportions, and p̄ = (p1 + p2)/2 = 0.39 is their average. For a two-sided significance level of 5%, Z1−α/2 = 1.96, and Z1−β = 0.84 was used to achieve 80% statistical power. Substituting these values gave 31.1 participants per group, which was rounded up to 32 per group, for a total of 64 participants.

Enrollment, allocation, and intervention

Eligible consenting patients were enrolled sequentially, and participants with odd-numbered enrollment records were assigned to arm A and those with even-numbered records to arm B, producing two equal groups; the enrollment sequence was applied by the study team at the time of enrollment. Before enrollment began, a one-time lottery determined which intervention was attached to each arm (10% urea ointment to arm A and hygiene advice alone to arm B), but it determined only the treatment label attached to the two predetermined sequences and did not randomize individual participants. Because assignment was deterministic and predictable, the design was non-randomized, allocation concealment was not used, and the study was open-label. Arm A applied 10% urea ointment (Equra 10%, Square Pharmaceuticals Ltd., Bangladesh) gently over the treatment field twice daily from the first day of radiotherapy until 14 days after completion, whereas arm B received skin hygiene advice alone, which constituted routine departmental practice in 2022 and consisted of avoidance of friction, tight clothing, and harsh soaps; use of a mild cleanser; and keeping the irradiated skin clean and dry, with no prophylactic topical agent applied to the treatment field. The same advice was given to participants in both arms, so the two arms differed in the twice-daily application of 10% urea ointment and in any skin care behavior associated with applying it. Hygiene advice alone was selected as the comparator because it was the customary departmental practice at the time and therefore represented the care participants would otherwise have received. The timing of application in relation to the daily radiotherapy fraction was not standardized, and adherence was assessed by verbal report at each weekly visit rather than by an objective measure, so adherence was not quantified and no adherence rate can be reported. The quantity of ointment applied, the source of supply of the product, the management of application after skin breakdown, the rescue treatments permitted in either arm, and product-related adverse effects such as irritation were not specified in the protocol or systematically recorded on the study proforma, and whether any enrollment number was skipped or whether any participant crossed over between arms was not documented. The number of patients screened and the reasons for exclusion before enrollment were also not recorded. All 64 enrolled participants were allocated in equal numbers to the two arms, completed the assessment four weeks after radiotherapy, and were analyzed in their allocated arm, with no participant excluded after allocation (Figure 1).

**Figure 1:**
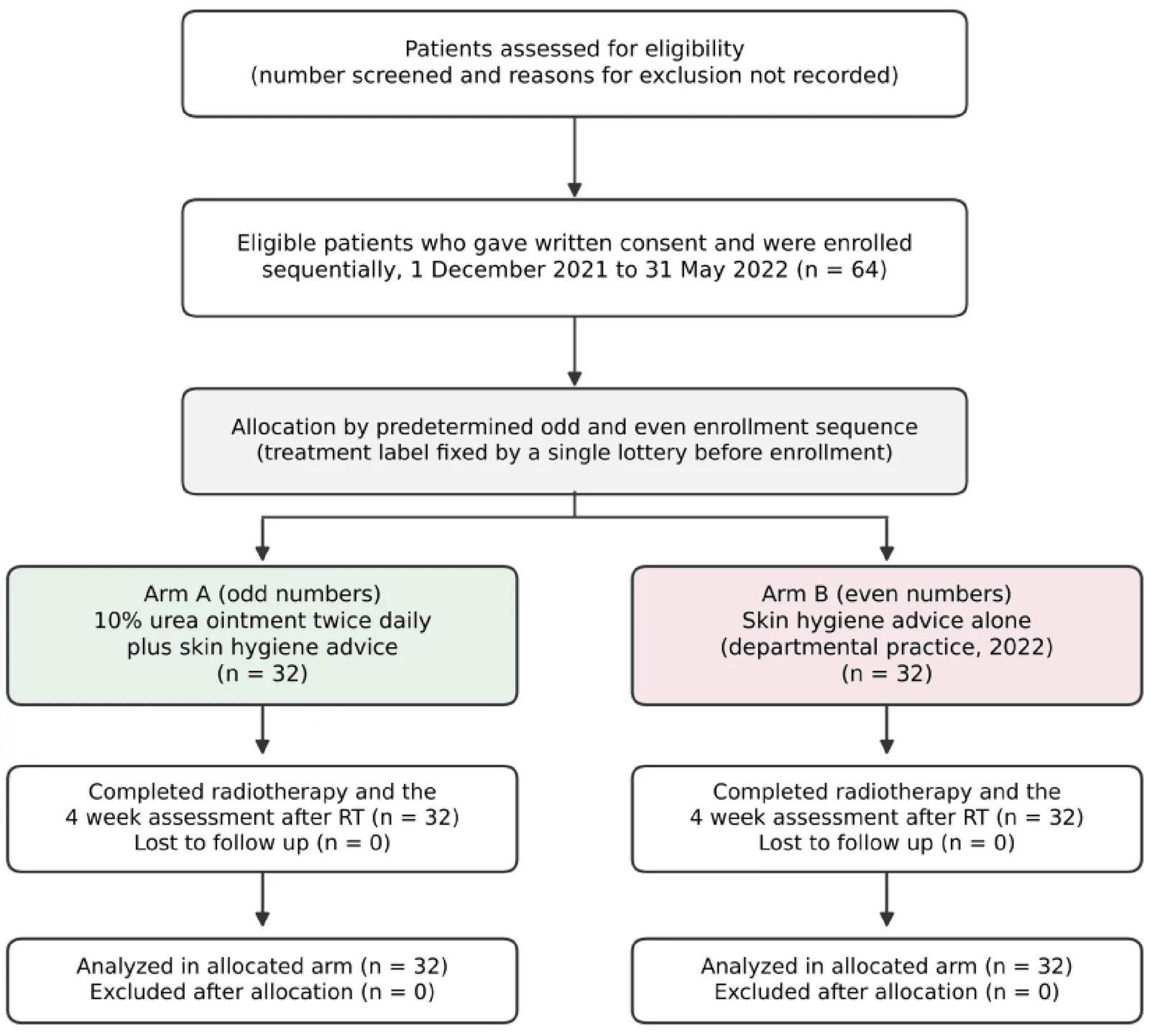
Flow of participants through the study. Allocation followed a predetermined odd-and-even enrollment sequence whose treatment labels were fixed by a single lottery before enrollment; individual participants were not randomized. The number of patients screened and the reasons for exclusion were not recorded. RT = radiotherapy

Outcomes and assessment

Patients were examined weekly during radiotherapy and at two and four weeks after completion. RISRs were graded using the RTOG acute skin toxicity criteria [6], in which grade 0 indicates no change; grade 1 follicular, faint or dull erythema, epilation, dry desquamation, or decreased sweating; grade 2 tender or bright erythema, patchy moist desquamation, or moderate edema; grade 3 confluent moist desquamation other than in skin folds or pitting edema; and grade 4 ulceration, hemorrhage, or necrosis. Grading was performed at the clinic visits by members of the study team who were aware of the allocation; the identity and training of the assessor at each visit were not documented, and no independent review of grading was available. Skin pain was scored on a 10-cm visual analog scale (VAS) [27]; whether each score was obtained before or after analgesic medication was not recorded. The primary outcome was the incidence of RISRs, defined as any RTOG grade ≥1 reaction within the treatment field at any scheduled assessment. Severe RISRs were defined as grade ≥3 at any scheduled assessment. For both cumulative outcomes, each participant was counted once, regardless of the number of assessments at which the reaction was present, whereas the weekly grade distributions are point prevalences in which the same participant may appear at several assessments. Secondary outcomes were the time from the first fraction to the first documented RISR; the duration of RISR; severe RISR; the intensity of skin pain; the need for analgesia (opioid or non-opioid); and the impact on treatment, classified as continued, interrupted, or discontinued. Treatment interruption was defined as any unplanned break in radiotherapy of at least one scheduled fraction, whatever its cause, and is therefore reported as all-cause unplanned radiotherapy interruption, and discontinuation as permanent cessation of radiotherapy before the prescribed dose had been delivered. The clinical reason for each interruption, the number and duration of breaks, the number of missed fractions, and the actual overall treatment time were not captured on the study proforma, and the indication, type, and timing of analgesia were not recorded beyond whether analgesia was required. For the time-to-event analysis, onset was assigned to the scheduled assessment at which a reaction was first documented, so the true onset lies between that visit and the previous one. The duration of RISR was derived from the dates of first appearance and of resolution documented at the scheduled visits and at any additional unscheduled attendance, which is why durations are reported in days rather than in whole weeks. Five participants (one in the urea arm and four in the hygiene advice arm) still had an active grade 1 reaction at the final assessment; because no resolution date within the study period was available for them, their durations extend only to the last documented assessment and are minimum, right-censored values.

Statistical analysis

Statistical analysis was performed using SPSS Statistics version 22 (IBM Corp., Armonk, NY, USA). Continuous variables were expressed as mean with standard deviation or median with range, while categorical variables were presented as frequencies and percentages. Between-group comparisons were performed using the independent-samples t test, Mann-Whitney U test, or Fisher’s exact test for two-by-two tables. Because several baseline and weekly grade tables contained sparse cells, multicategory distributions were compared with the Fisher Freeman-Halton exact test, and ordered RTOG grades with an exact permutation version of the linear-by-linear association (Cochran-Armitage) trend test, both computed from the cross-tabulated counts. Baseline balance was additionally described by absolute standardized differences, with values above 0.10 taken to indicate meaningful imbalance, because significance tests in a sample of this size do not establish comparability. Unadjusted risk ratios (RRs) and absolute risk differences (ARDs) with 95% confidence intervals (CIs) were estimated for selected clinical outcomes. Exploratory adjusted RRs were obtained by modified Poisson regression with robust standard errors, adjusting for socioeconomic status, age, and sex, with tumor site (head and neck versus other) added in a sensitivity model. These models were not specified in a dated public protocol and are therefore regarded as exploratory. Socioeconomic status was entered as poor versus middle or upper class, because only one participant was upper class. All models were fitted to the full sample of 64 participants, as there were no missing values for the outcomes or covariates. Time to the first documented RISR was analyzed using the Kaplan-Meier method and log-rank test, with the scheduled assessment number as the time scale and reaction-free participants censored at the final assessment. Week-specific comparisons were exploratory and were not adjusted for multiplicity. A two-sided p-value <0.05 was considered statistically significant.

Ethics

The study protocol was reviewed and approved by the Ethical Review Committee of Dhaka Medical College, Dhaka, Bangladesh (memo number: ERC-DMC/ECC/2021/411). The study was conducted in accordance with the ethical principles of the Declaration of Helsinki, and written informed consent was obtained from every participant before enrollment, after the purpose of the study, the procedures involved, and the voluntary nature of participation had been explained.

## Results

The mean age was 52.8 ± 12.5 years in the urea arm and 55.0 ± 10.9 years in the hygiene advice arm. Although age, sex, educational level, smoking history, comorbidity, and Eastern Cooperative Oncology Group Performance (ECOG) performance status did not differ significantly, the absolute standardized difference exceeded 0.10 for every characteristic except ECOG performance status, so the absence of significant differences should not be taken as evidence of clinical comparability. The largest imbalance concerned socioeconomic status (standardized difference = 1.17). In the hygiene advice arm, 24 of the 32 patients (75.0%) were poor compared with 8 (25.0%) in the urea arm, while 23 (71.9%) in the urea arm were middle class compared with 8 (25.0%) in the hygiene advice arm (p < 0.001). Educational level also differed appreciably (standardized difference = 0.66), with more illiterate participants in the hygiene advice arm (Table 1).

**Table 1:** Baseline demographic and clinical characteristics of participants by treatment arm. Values are n (%) unless otherwise stated. a: Fisher-Freeman-Halton exact test; b: Fisher’s exact test; c: independent-samples t-test. Standard difference: absolute standardized difference, calculated for multicategory variables across all categories; values above 0.10 indicate meaningful imbalance. SSC = Secondary School Certificate; HSC = Higher Secondary Certificate; ECOG = Eastern Cooperative Oncology Group

Characteristic	10% urea (n = 32)	Hygiene advice alone (n = 32)	P-value	Standard difference
Age (years), mean ±SD	52.8 ±12.5	55.0 ± 10.9	0.46^c^	0.19
Sex			0.44^b^	0.26
Male	18 (56.3)	22 (68.8)		
Female	14 (43.8)	10 (31.3)		
Socioeconomic status			<0.001^a^	1.17
Poor	8 (25.0)	24 (75.0)		
Middle class	23 (71.9)	8 (25.0)		
Upper class	1 (3.1)	0 (0.0)		
Education			0.10^a^	0.66
Illiterate	4 (12.5)	11 (34.4)		
Under SSC	16 (50.0)	15 (46.9)		
SSC	7 (21.9)	5 (15.6)		
HSC	5 (15.6)	1 (3.1)		
Smoking history			0.50^a^	0.30
Never	11 (34.4)	10 (31.3)		
Current	14 (43.8)	18 (56.3)		
Former	7 (21.9)	4 (12.5)		
Comorbidity			0.33^a^	0.45
None	20 (62.5)	18 (56.3)		
Hypertension	12 (37.5)	11 (34.4)		
Cardiovascular disease	0 (0.0)	3 (9.4)		
ECOG performance status			1.00^a^	0.00
0	22 (68.8)	22 (68.8)		
1	8 (25.0)	8 (25.0)		
2	2 (6.3)	2 (6.3)		

Head and neck cancers predominated in both arms but were more frequent in the hygiene advice arm (22 of 32, 68.8%) than in the urea arm (16, 50.0%), a standardized difference of 0.39 for head and neck versus other sites, whereas gynecological cancers were more frequent with urea (10/32, 31.3% versus 6/32, 18.8%). None of the disease characteristics differed significantly, but the standardized differences were 0.41 for tumor site, 0.36 for stage, 0.31 for histology, and 0.14 for treatment intent. Because the irradiated site influences the severity of skin reactions, tumor site was added as a covariate in a sensitivity analysis. Most patients had stage II or III squamous cell carcinoma. The prescribed dose, fractionation, field size, bolus use, and overall treatment time were not recorded on the study proforma and therefore could not be compared between the arms (Table 2).

**Table 2:** Baseline disease and radiotherapy characteristics of participants by treatment arm. Values are n (%) unless otherwise stated. a: Fisher-Freeman-Halton exact test; b: Fisher’s exact test. Standard difference: absolute standardized difference (0.39 for head and neck versus other sites). Prescribed and delivered dose, dose per fraction, number of fractions, field size, skin location within the field, bolus use, and planned and actual treatment duration were not captured on the study proforma. RT = radiotherapy

Characteristic	10% urea (n = 32)	Hygiene advice alone (n = 32)	P-value	Standard difference
Tumor site			0.58^a^	0.41
Head and neck	16 (50.0)	22 (68.8)		
Gynecological	10 (31.3)	6 (18.8)		
Gastrointestinal	4 (12.5)	2 (6.3)		
Genitourinary	1 (3.1)	1 (3.1)		
Thoracic	1 (3.1)	1 (3.1)		
Disease stage			0.63^a^	0.36
I	2 (6.3)	1 (3.1)		
II	10 (31.3)	13 (40.6)		
III	20 (62.5)	17 (53.1)		
IV	0 (0.0)	1 (3.1)		
Histology			0.51^a^	0.31
Squamous cell carcinoma	27 (84.4)	30 (93.8)		
Adenocarcinoma	2 (6.3)	1 (3.1)		
Others	3 (9.4)	1 (3.1)		
RT intent, definitive/Postoperative	25 / 7	23 / 9	0.77^b^	0.14

No reactions occurred in either arm during the first week, and only a few appeared in the second. From the third week onward, a higher proportion of the hygiene advice arm had active reactions. The grade distribution differed at the third week (exact p = 0.004), reflecting the lower overall frequency of reactions with urea rather than greater severity, as the trend test was not significant at that assessment (exact trend p = 0.16), and it differed again at week six or completion (exact p = 0.006). At week six/completion, active reactions were present in 22/32 (68.8%) of the hygiene advice arm versus 11/32 (34.4%) of the urea arm; 21 of the 32 urea-arm patients remained grade 0 compared with 10 of 32 with hygiene advice alone; grade 2 or worse was recorded in 3 versus 16 patients; and the only grade 4 reaction occurred with hygiene advice alone. The exact trend test indicated higher grades in the hygiene advice arm at the fifth week (p = 0.033) and at week six/completion (p < 0.001), but not at the second, third, or fourth week or at either assessment after radiotherapy. Reactions receded after radiotherapy in both arms, and by the fourth post-radiotherapy week only one urea arm and four hygiene advice patients still had an active reaction, all grade 1 (Table 3).

**Table 3:** Distribution of RTOG acute skin toxicity grades by treatment arm at each scheduled assessment. Values are n (%) of the 32 patients in each arm at the stated assessment (point prevalence); percentages may not sum to 100 because of rounding. A participant may contribute to several assessments, so weekly counts cannot be added to obtain cumulative outcomes (see Table 4). *: p for the between-arm difference in the full distribution of RTOG grades (Fisher-Freeman-Halton exact test). †: p for the exact linear-by-linear (Cochran-Armitage) trend toward higher grades. All weekly tests are exploratory and were not adjusted for multiplicity. Hygiene advice, hygiene advice alone. RTOG = Radiation Therapy Oncology Group; RT = radiotherapy

Assessment	Arm	Grade 0	Grade 1	Grade 2	Grade 3	Grade 4	p^*^	p^†^
Week 1	10% urea	32 (100.0)	0 (0.0)	0 (0.0)	0 (0.0)	0 (0.0)	-	-
	Hygiene advice	32 (100.0)	0 (0.0)	0 (0.0)	0 (0.0)	0 (0.0)		
Week 2	10% urea	27 (84.4)	5 (15.6)	0 (0.0)	0 (0.0)	0 (0.0)	0.71	1.00
	Hygiene advice	28 (87.5)	3 (9.4)	1 (3.1)	0 (0.0)	0 (0.0)		
Week 3	10% urea	26 (81.2)	3 (9.4)	1 (3.1)	2 (6.2)	0 (0.0)	0.004	0.16
	Hygiene advice	16 (50.0)	11 (34.4)	5 (15.6)	0 (0.0)	0 (0.0)		
Week 4	10% urea	17 (53.1)	10 (31.2)	4 (12.5)	1 (3.1)	0 (0.0)	0.81	0.47
	Hygiene advice	14 (43.8)	10 (31.2)	7 (21.9)	1 (3.1)	0 (0.0)		
Week 5	10% urea	17 (53.1)	11 (34.4)	3 (9.4)	1 (3.1)	0 (0.0)	0.15	0.033
	Hygiene advice	11 (34.4)	10 (31.2)	5 (15.6)	6 (18.8)	0 (0.0)		
Week 6/Completion	10% urea	21 (65.6)	8 (25.0)	1 (3.1)	2 (6.2)	0 (0.0)	0.006	<0.001
	Hygiene advice	10 (31.2)	6 (18.8)	7 (21.9)	8 (25.0)	1 (3.1)		
2 weeks post-RT	10% urea	27 (84.4)	3 (9.4)	2 (6.2)	0 (0.0)	0 (0.0)	0.094	0.064
	Hygiene advice	19 (59.4)	8 (25.0)	5 (15.6)	0 (0.0)	0 (0.0)		
4 weeks post-RT	10% urea	31 (96.9)	1 (3.1)	0 (0.0)	0 (0.0)	0 (0.0)	0.35	0.35
	Hygiene advice	28 (87.5)	4 (12.5)	0 (0.0)	0 (0.0)	0 (0.0)		

On time-to-event analysis with the scheduled assessment as the time scale, RISR was first documented at a later assessment with urea (log-rank p = 0.024). The cumulative incidence in the hygiene advice arm reached 16/32 (50%) by the third assessment and 24/32 (75%) by the end of follow-up, whereas with urea it rose more slowly and plateaued at 15/32 (46.9%) from the fourth assessment onward. Among affected participants only, the first reaction was documented at a median of week four with urea and week three with hygiene advice alone; this is a conditional descriptive statistic rather than the median event time of the full cohort. Because the time scale reflects assessment number rather than elapsed time or delivered fractions, which may differ when interruptions differ between arms, this finding should be regarded as provisional. Cumulative incidence curves with 95% CIs and numbers at risk are shown in Figure 2.

**Figure 2:**
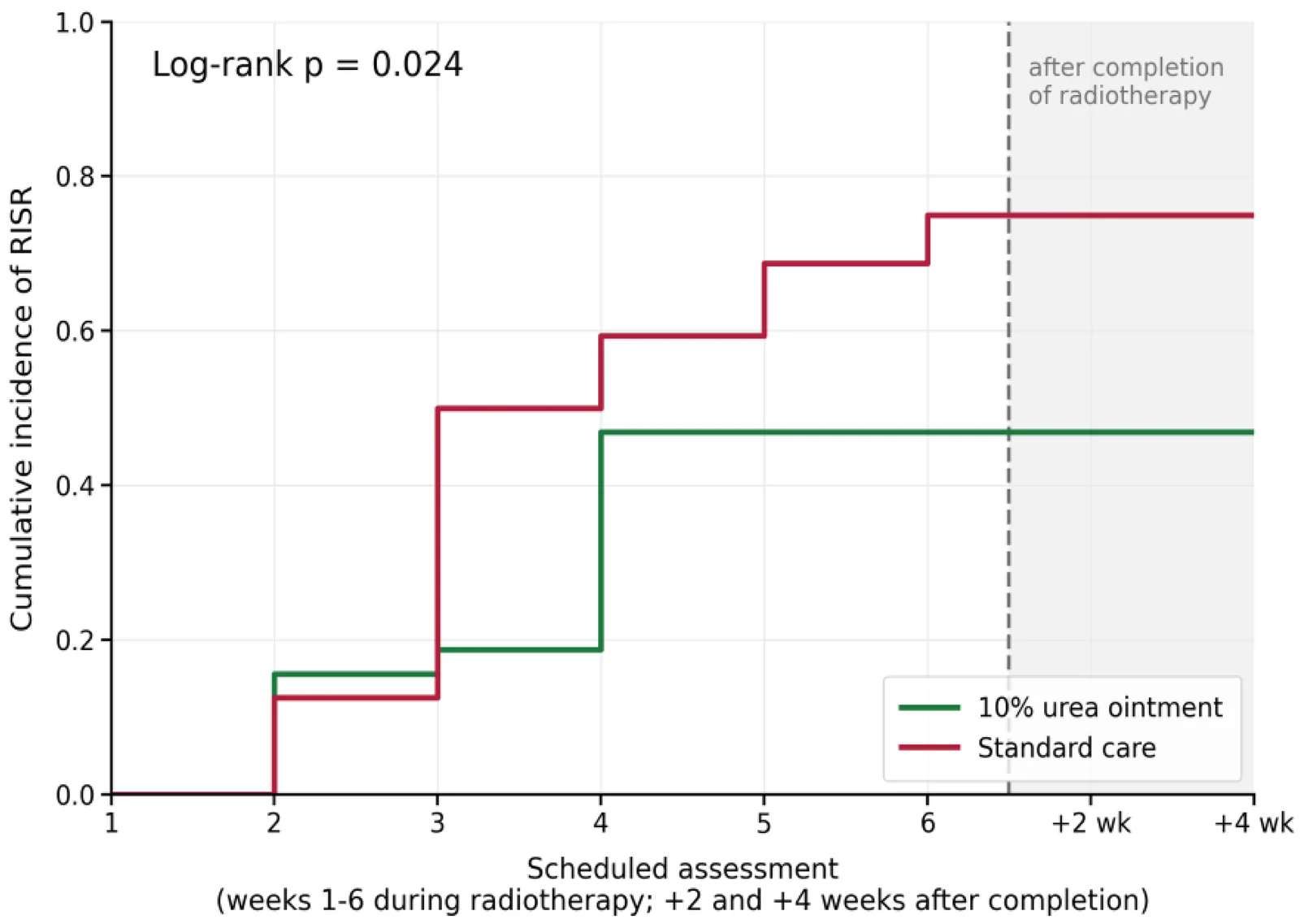
Unadjusted cumulative incidence (one minus the Kaplan-Meier estimate of remaining reaction-free) of radiation-induced skin reactions by scheduled assessment in the two arms. The horizontal axis shows the scheduled assessments: weeks 1-6 during radiotherapy, then 2 and 4 weeks after its completion (shaded). Reaction-free patients were censored at the end of follow-up; groups were compared by the log-rank test.

Over the treatment and follow-up period, RISR occurred in 15/32 (46.9%) of the urea arm and 24/32 (75.0%) of the hygiene advice arm (p = 0.039). Severe (grade ≥3) reactions were less frequent with urea (6/32, 18.8% versus 10/32, 31.2%) but not significantly. Considering the maximum RTOG grade of each participant over the whole observation period, 17 (53.1%) urea and 8 (25.0%) hygiene advice participants never developed a reaction, 9 (28.1%) and 14 (43.8%) reached a maximum of grade 1 or 2, 6 (18.8%) and 9 (28.1%) reached grade 3, and none and 1 (3.1%) reached grade 4 (exact p = 0.094). Among affected participants, median duration was 15.0 days (range = 8-29) versus 21.5 days (range = 7-39; p = 0.082); this numerical difference was not statistically significant, includes five censored durations, and is conditional on developing a reaction, so it is descriptive only. Analgesia was required by 9/32 (28.1%) and 19/32 (59.4%), respectively (p = 0.023). All-cause unplanned radiotherapy interruption occurred in 4/32 (12.5%) of the urea arm and 22/32 (68.8%) of the hygiene advice arm (p < 0.001), and no patient in either arm discontinued treatment permanently (Table 4).

**Table 4:** Comparison of primary and secondary clinical outcomes between the 10% urea and hygiene advice alone arms. +: Among patients who developed RISR (urea n = 15, standard n = 24); p from Mann-Whitney U test. Other p-values from Fisher’s exact test. The p-value for the impact on treatment is for the comparison of interrupted versus not interrupted; no participant discontinued radiotherapy. RISR = radiation-induced skin reaction; VAS = visual analog scale

Outcome	10% urea (n=32)	Hygiene advice alone (n=32)	p-value
Any RISR at any assessment, n (%)	15 (46.9)	24 (75.0)	0.039
Severe (grade ≥3) RISR at any assessment, n (%)	6 (18.8)	10 (31.2)	0.39
Maximum RTOG grade over follow up, n (%)			
Grade 0 (no reaction)	17 (53.1)	8 (25.0)	0.094
Grade 1 or 2	9 (28.1)	14 (43.8)	
Grade 3	6 (18.8)	9 (28.1)	
Grade 4	0 (0.0)	1 (3.1)	
Median duration of RISR, days (range)^+^	15.0 (8-29)	21.5 (7-39)	0.082
Analgesia required, n (%)	9 (28.1)	19 (59.4)	0.023
Impact on treatment			
Continued without interruption	28 (87.5)	10 (31.2)	<0.001
All cause unplanned interruption	4 (12.5)	22 (68.8)	
Discontinued	0 (0.0)	0 (0.0)	

Weekly skin pain scores at each assessment are shown in Table 5. Group mean VAS scores never exceeded 2.2 of a possible 10 at any assessment, rose gradually to a maximum at week six or on completion of radiotherapy and fell thereafter, and did not differ significantly between the arms at any of the eight scheduled assessments. Individual scores were nevertheless higher in some participants, reaching 10 in one urea arm participant at week four and seven in the hygiene advice arm at week five, so low group means do not imply that pain was mild in every patient. The largest difference was recorded at week six/completion, where the mean score was 1.50 ± 1.67 in the urea arm and 2.19 ± 1.64 in the hygiene advice arm (p = 0.068).

**Table 5:** Comparison of skin pain intensity between treatment arms at each scheduled assessment. Scores range from 0 (no pain) to 10 (worst imaginable pain). p from the Mann-Whitney U test. Weekly comparisons are exploratory repeated measurements on the same patients and were not adjusted for multiple testing. Individual scores reached 10 in one participant, and whether each score was recorded before or after analgesic medication was not documented. SD = standard deviation; RT = radiotherapy

Assessment	10% urea (n = 32)		Hygiene advice alone (n = 32)		P-value
	Mean ± SD	Median (range)	Mean ± SD	Median (range)	
Week 1	0.12 ± 0.55	0 (0–3)	0.00 ± 0.00	0 (0–0)	0.16
Week 2	0.41 ± 1.04	0 (0–4)	0.41 ± 0.91	0 (0–4)	0.47
Week 3	1.00 ± 1.68	0 (0–7)	0.75 ± 1.05	0 (0–4)	0.98
Week 4	1.44 ± 1.97	1 (0–10)	1.38 ± 1.56	1 (0–4)	0.98
Week 5	1.59 ± 1.56	1 (0–5)	1.88 ± 1.70	1 (0–7)	0.51
Week 6/Completion	1.50 ± 1.67	1.5 (0–6)	2.19 ± 1.64	2 (0–6)	0.068
2 weeks post-RT	0.72 ± 1.28	0 (0–5)	0.69 ± 0.82	0 (0–2)	0.48
4 weeks post-RT	0.19 ± 0.40	0 (0–1)	0.22 ± 0.42	0 (0–1)	0.77

The unadjusted and adjusted RRs are shown in Table 6. In unadjusted analyses, the risk of any RISR was 38% lower with urea (RR = 0.62, 95% CI = 0.41-0.95; absolute risk difference = 28.1%, 95% CI = 5.2-51.0), the risk of requiring analgesia 53% lower (RR = 0.47, 95% CI = 0.25-0.88), and the risk of all-cause unplanned interruption 82% lower (RR = 0.18, 95% CI = 0.07-0.47). Grade ≥3 reactions showed the same direction but were not significant (RR = 0.60, 95% CI = 0.25-1.45). In the exploratory model adjusted for socioeconomic status, age and sex, the associations with any RISR (adjusted RR = 0.62, 95% CI = 0.41-0.93), and interruption (adjusted RR = 0.29, 95% CI = 0.11-0.79) remained significant, whereas the reduction in analgesia use was no longer significant (adjusted RR = 0.55, 95% CI = 0.27-1.11). Adding tumor site to the model changed these estimates little (any RISR = 0.67, 95% CI = 0.45-0.98; interruption = 0.29, 95% CI = 0.11-0.80; analgesia use = 0.57, 95% CI = 0.28-1.17). 

**Table 6:** Unadjusted and adjusted risk estimates for key clinical outcomes in the 10% urea (n = 32) and standard care (n = 32) arms. RRs below 1 indicate a lower observed risk in the 10% urea arm. Unadjusted p-values are from Fisher’s exact test; adjusted RRs are from exploratory modified Poisson regression (robust variance) fitted to all 64 participants with a: socioeconomic status (poor versus middle or upper class), age and sex as covariates and b: socioeconomic status, age, sex and tumor site (head and neck versus other) as covariates. NE, not estimated (only 16 grade ≥3 events (6 with urea, 10 with hygiene advice alone), too few for stable multivariable adjustment). Interruption denotes all cause unplanned interruption, whose reasons were not recorded. With few events per covariate, robust variance intervals may understate uncertainty. Because allocation was non-random, these estimates do not establish causality. RISR = radiation-induced skin reaction; RT = radiotherapy; RR = risk ratio; CI = confidence interval; ARD = absolute risk difference (hygiene advice alone minus 10% urea); a positive value favors urea; RT = radiotherapy

Outcome	Unadjusted RR (95% CI)	Adjusted RR (95% CI)^a^	Adjusted RR (95% CI)^b^	ARD, % (95% CI)	P-value
Any RISR	0.62 (0.41-0.95)	0.62 (0.41-0.93)	0.67 (0.45-0.98)	28.1 (5.2, 51.0)	0.039
Severe (grade ≥3) RISR	0.60 (0.25-1.45)	NE	NE	12.5 (-8.5, 33.5)	0.39
Analgesia use	0.47 (0.25-0.88)	0.55 (0.27-1.11)	0.57 (0.28-1.17)	31.2 (8.2, 54.3)	0.023
All cause unplanned RT interruption	0.18 (0.07-0.47)	0.29 (0.11-0.79)	0.29 (0.11-0.80)	56.2 (36.5, 76.0)	<0.001

Both the unadjusted estimates and those from the sensitivity model adjusted for socioeconomic status, age, sex, and tumor site are displayed as a forest plot in Figure 3, in which RRs below one favor urea and CIs excluding one denote statistically significant associations.

**Figure 3:**
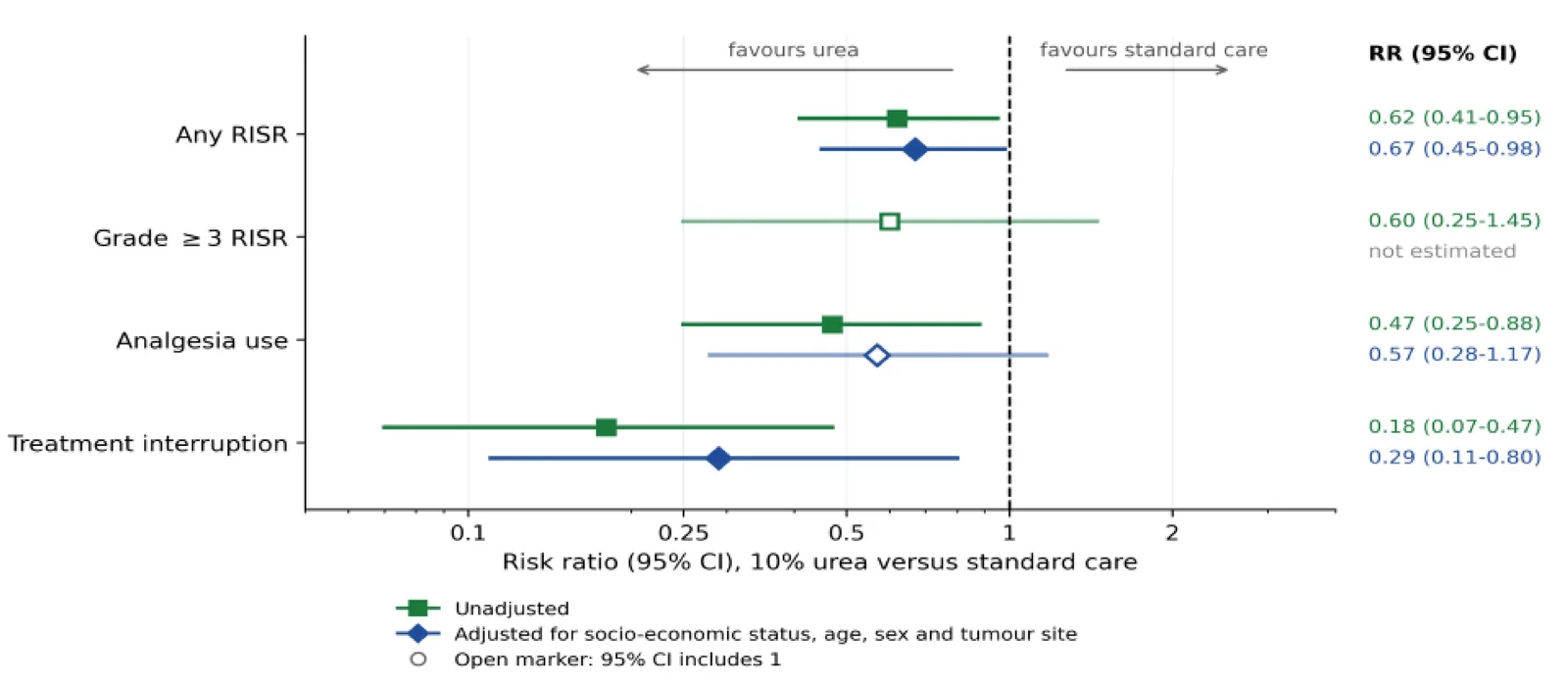
Forest plot of unadjusted and adjusted RRs (95% CI) for the principal outcomes. RRs below 1 indicate lower observed risk in the 10% urea arm; adjusted estimates are from exploratory modified Poisson regression with robust variance including socioeconomic status, age, sex, and tumor site, and an open marker denotes a 95% CI that includes 1. The adjusted estimate for grade ≥3 reactions was not calculated because of the small number of events. Interruption denotes all cause unplanned RT interruption, whose reasons were not recorded. RT = radiotherapy; RR = risk ratio; CI = confidence interval

## Discussion

The present study indicated that adding twice-daily 10% urea ointment to departmental skin hygiene advice was associated with a lower incidence of RISRs than hygiene advice alone. Reactions were recorded in 15/32 (46.9%) of the urea arm and 24/32 (75.0%) of the hygiene advice arm and were first documented at later assessments, although this temporal finding rests on an assessment-based time scale and is provisional. Severity favored urea at some but not all weekly assessments, and the shorter median duration of reactions (15.0 versus 21.5 days) was a numerical difference that was not statistically significant. Fewer patients in the urea arm required analgesia or had an all-cause unplanned interruption of radiotherapy, whereas mean skin pain scores were low throughout and did not differ at any assessment.

Consistent with our results, a systematic review and meta-analysis concluded that proactive moisturization reduces acute radiation dermatitis and improves skin-related quality of life, albeit with weak-to-moderate certainty [20], and meta-analyses of topical non-steroidal [21] and of natural or miscellaneous agents [19] reached similar conclusions. The separation of the incidence curves from the third week is consistent with the natural history of these reactions, which appear within one to four weeks of starting radiotherapy and progress as the cumulative dose rises [3,4]. That the curves continued to diverge suggests the difference between the arms widened as treatment proceeded.

In contrast to our study, a randomized trial of herbal creams in breast cancer radiotherapy found neither reduced severity nor delayed onset of dermatitis [28], indicating that a moisturizing vehicle alone does not guarantee benefit. Such differences between studies may reflect variation in the active agent and its formulation, the irradiated site and dose, the fractionation schedule, and the grading instrument, all of which influence reported reaction rates and complicate direct comparison [6,14]. Our participants were treated on a cobalt-60 machine, largely for head and neck cancer, unlike the breast cancer populations of earlier work.

A protective association with urea is biologically plausible, because urea is a humectant that binds water within the stratum corneum and supports the epidermal barrier [4,5], which might raise the cumulative dose at which clinically evident desquamation appears. However, barrier function, skin hydration, and inflammatory markers were not measured, and the study had no vehicle-only or alternative moisturizer arm. The observed contrast therefore reflects the addition of a urea-containing ointment, together with any change in skin care behavior that its twice-daily application entailed, and cannot distinguish an effect specific to urea from the general effect of adding moisturization [7,16]. Other measures such as barrier films or avoidance of antiperspirants act through different pathways [11,29]. Because guidelines recommend gentle washing together with moisturization [15,17,18], our comparator reflected the hygiene component of that recommendation but omitted the moisturizing component, so the study does not establish superiority of urea over other moisturizing regimens or over a fully guideline-concordant regimen.

Fewer participants in the urea arm had an all-cause unplanned interruption of radiotherapy (4/32, 12.5% versus 22/32, 68.8%), and no participant in either arm stopped treatment permanently. Because prolonged overall treatment time can compromise local tumor control, a difference of this size would be clinically important if it were confirmed to be skin-related [2,6]. It is, however, larger than the difference in severe skin toxicity would predict, and because the reason, number and duration of breaks, the missed fractions, and the overall treatment time were not recorded, skin-related breaks cannot be separated from those due to machine downtime, holidays, financial difficulty, or tumor symptoms. The finding therefore cannot be attributed to the prevention of skin toxicity, is the least secure of our results, and needs confirmation with prospectively recorded, reason-specific data.

Our study adds preliminary data on an inexpensive, locally available agent in a South Asian population under-represented in previous prophylaxis trials, most of which studied breast cancer patients in high-income countries [8,30]. In Bangladesh, where radiotherapy capacity is limited, most patients attending public cancer centers are poor and out-of-pocket spending is a recognized source of hardship [22-25]; an agent that costs little and may help patients complete curative radiotherapy would be of practical value if confirmed.

The present study has several strengths. To our knowledge, it is the first prospective evaluation of prophylaxis against RISRs conducted in Bangladesh; it tested a low-cost, widely available agent against the care patients would otherwise have received, applied a standardized RTOG assessment weekly during radiotherapy and at two and four weeks afterward, achieved complete follow-up in all 64 participants, and reported incidence, onset, duration, severity, pain, analgesia use, and treatment continuity rather than a single endpoint.

Several limitations also apply. Participants were assigned by a predictable odd-even sequence rather than randomized, allocation was not concealed, and the study was open-label, with grading by assessors aware of allocation and without independent review, so selection, performance, and observer bias cannot be excluded. Institutional ethical clearance was obtained on November 23, 2021, before the first participant was enrolled, but the study was not registered in a public clinical trial registry at any stage, and no dated statistical analysis plan is available. The reported outcomes and adjusted models therefore cannot be checked against a prespecified public record, and the possibility of outcome reporting bias cannot be formally excluded. Baseline imbalance, particularly in socioeconomic status and education, indicates incomplete exchangeability. Tumor site could be adjusted for only as a binary covariate (head and neck versus other), and more detailed site-specific modeling, as well as adjustment for dose, fractionation, irradiated volume, and bolus use, was not feasible because the sample was small and these radiotherapy characteristics were not recorded. With few events per covariate, robust variance CIs may understate uncertainty. The reason, number, and duration of interruptions; the missed fractions; and the overall treatment time were not recorded, which limits interpretation of the largest observed difference. Onset was known only to the nearest scheduled assessment; the time scale did not represent elapsed time or delivered fractions; five reaction durations were censored at the final assessment; and the duration comparison was restricted to participants who developed a reaction. Adherence was assessed only by verbal report and was not quantified; the quantity applied and rescue treatments were not standardized; and tolerability of the ointment was not systematically recorded. The comparator, although it reflected the skin hygiene advice that guidelines recommend, did not include the prophylactic moisturization that guidelines also endorse, and there was no vehicle arm. Week-specific comparisons were exploratory, and only acute reactions were assessed. Confirmation requires a randomized, outcome-assessor-blinded multicenter trial stratified by treatment site, with recorded radiotherapy parameters, a vehicle or alternative moisturizer comparator, and prospectively recorded reasons for interruption.

## Conclusions

In this small, non-randomized study, adding twice-daily 10% urea ointment to departmental skin hygiene advice was associated with fewer observed RISRs, which were also first documented at later assessments. Analgesia use was lower in unadjusted analysis but did not survive covariate adjustment, weekly skin pain scores did not differ, and the shorter reaction duration was not statistically significant. The lower rate of all-cause unplanned treatment interruption requires cautious interpretation because the reasons for interruption were not recorded. Because allocation was deterministic and baseline characteristics were imbalanced, these estimates cannot establish causality and do not establish superiority over other moisturizing regimens. Urea remains a low-cost, locally available candidate for RISR prophylaxis in resource-limited settings, and confirmation requires an adequately powered randomized multicenter trial stratified by treatment site and using blinded outcome assessment.
